# Gene expression-based screening for inhibitors of PDGFR signaling

**DOI:** 10.1186/gb-2008-9-3-r47

**Published:** 2008-03-01

**Authors:** Alena A Antipova, Brent R Stockwell, Todd R Golub

**Affiliations:** 1 Cancer Program, Broad Institute of Massachusetts Institute of Technology and Harvard University, Cambridge Center, Cambridge, MA 02142, USA; 2 Department of Pediatric Oncology, Dana-Farber Cancer Institute and Harvard Medical School, Binney Street, Boston, MA 02115, USA; 3 Department of Chemistry, Massachusetts Institute of Technology, Massachusetts Avenue, Cambridge, MA 02139, USA; 4 Departments of Biological Sciences and Chemistry, Columbia University, Fairchild Center MC2406, Amsterdam Avenue, New York, NY 10027, USA; 5 Howard Hughes Medical Institute, Jones Boulevard, Chevy Chase, MD 20815, USA; 6 Current address: Advanced Genetic Analysis, Applied Biosystems, Cummings Center, Beverly, MA 01915, USA

## Abstract

Here we describe a proof-of-concept experiment designed to explore the possibility of using gene expression-based high-throughput screening (GE-HTS) to find inhibitors of a signaling cascade, using platelet derived growth factor receptor (PDGFR) signaling as the example. The previously unrecognized ability of aurintricarboxylic acid to inhibit PDGFR signaling, discovered through a screen of 1,739 compounds, demonstrates the feasibility and generalizability of GE-HTS for the discovery of small molecule modulators of any signaling pathway of interest.

## Abstract 2

Inhibitors of the platelet derived growth factor receptor (PDGFR) signaling pathway are isolated using gene expression-based high-throughput screening (GE-HTS), a method that is applicable to other pathways.

## Background

High throughput screening of small-molecule libraries is a well-established and highly productive tool for the identification of chemical compounds targeting a specific protein function of interest. Traditionally, the high-throughput screening for modulators of molecular pathways involves cell-free biochemical assays, or in some cases, highly specialized cell-based phenotypic assays [1]. However, in many cases the optimal target for therapeutic intervention is not known, or the development of a suitable phenotypic read-out is not technically feasible. For example, it is becoming increasingly of interest to modulate the activity of particular signal transduction pathways, but the components of such pathways are in many cases only partially known. It would therefore be of interest to develop a screening approach that could identify inhibitors of such pathways without first defining the biochemical target of candidate small molecules. Here we demonstrate that it is possible to use mRNA expression levels as a read-out to infer activity of a signal transduction pathway, thus establishing a general approach to screening for modulators of signal transduction pathways.

Endogenous mRNA expression has been previously successfully used as a surrogate of cellular states in high-throughput screening for compounds inducing differentiation of acute myeloid leukemia cells, and for identifying inhibitors of androgen receptor-mediated transcriptional activation in prostate cancer [2-5]. It is not obvious, however, that gene expression signatures could be used to identify inhibitors of signal transduction pathways that are regulated at the level of post-translational modification (phosphorylation), as opposed to transcriptional regulation.

To test the feasibility of using gene expression-based high-throughput screening (GE-HTS) to identify inhibitors of a signaling pathway, we chose platelet derived growth factor receptor (PDGFR) signaling for a proof-of-concept study, with particular emphasis on downstream activation of the extracellular regulated kinase (ERK) pathway (also known as the p42/p44 mitogen activated protein (MAP) kinase pathway) as a target pathway for the screen. The ERK pathway plays a major role in the control of cell growth, cell differentiation and cell survival [6]. The protein kinase cascade Raf>mitogen/extracellular signal-regulated kinase (MEK)>ERK, also referred to as the MAP kinase module, is activated in mammalian cells through receptor tyrosine kinases, G-protein coupled receptors and integrins [6]. Activated ERKs translocate to the nucleus where they phosphorylate transcription factors. The ERK pathway is often upregulated in human tumors [6], and as such is an attractive target for anticancer therapy. Furthermore, because the pathway has been extensively studied, many experimental tools are available with which to interrogate the pathway. We demonstrate here that indeed small molecule inhibitors of the PDGFR/ERK pathway can be discovered using the GE-HTS approach.

## Results

### Identification of a signature of PDGFR/ERK activity

**Figure 1:**
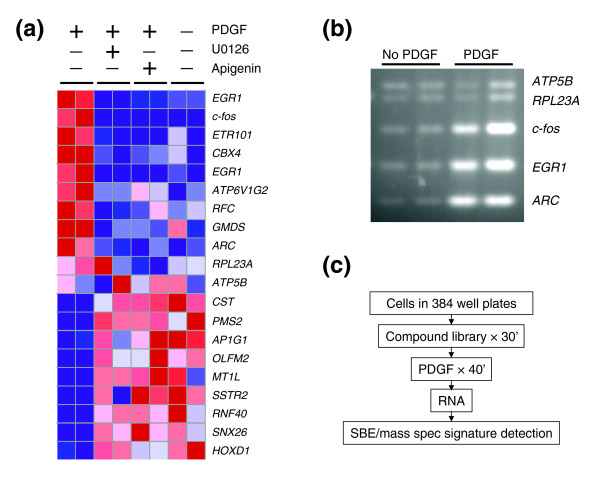
PDGFR/ERK activation signature for high-throughput screening. **(a) **Genes whose expression is correlated with ERK activation by PDGFR. Genes (in rows) sorted by their expression in samples (columns) with or without U0126, apigenin, and PDGF. Red indicates high relative expression, blue low expression. **(b) **RT-PCR of signature genes in four sample wells: two lanes (replicas) per condition. TIP5 cells were serum starved overnight and then treated with PDGF. **(c) **Screening schema overview. SBE, single-base extension.

In GE-HTS, a gene expression signature is used as a surrogate of a biological state. In the present context, we sought to define a signature of ERK activation mediated by PDGFR stimulation. Specifically, we treated SH-SY5Y neuroblastoma cells with the BB homodimer of PDGF (PDGF-BB), which resulted in PDGFRβ phosphorylation and subsequent ERK activation. We selected PDGFRβ over PDGFRα for our studies because of previous observations that PDGFRα might mediate functions of other PDGF isoforms in addition to PDGF-A [7,8]. The activation state of the members of the PDGFβ pathway can be traced by increase in their phosphorylation levels shortly after introduction of the growth factor [9]. In particular, ERK phosphorylation peaks at about 15-20 minutes after induction, and then decreases to background levels some 20-30 minutes later [10]. Accordingly, we performed gene expression profiling using Affymetrix U133A arrays 30 minutes following PDGF stimulation, thereby identifying those genes whose expression is correlated with PDGFR activity. In order to identify the component of the gene expression signature that was attributable to ERK activation by PDGFR (as opposed to other pathways downstream of PDGFR), we also pretreated the cells with the MEK inhibitor U0126 and the ERK inhibitor apigenin, and repeated the gene expression profiling studies (Figure 1a).

To define the signature of ERK activation, we developed and applied a rank-pairwise comparison algorithm as described in Materials and methods. We note that the genes identified in this manner are chosen because of their ability to reflect the PDGF-stimulated state - not because of their necessarily being critical effectors of PDGFR signaling. The top three genes identified in this fashion were those for c-fos, early growth response 1 (*EGR1*), and activity-regulated cytoskeleton-associated protein (*ARC*). All three genes were previously shown to be upregulated by activation of ERK, and we further confirmed their regulation by reverse transcriptase (RT)-PCR (Figure 1b) [11-13]. Two additional genes, ribosomal protein *RPL23A *and *ATP5B*, were selected as internal controls, because their expression was not significantly altered by PDGFR activation.

### High-throughput screening to find inhibitors of the PDGFR/ERK pathway

Having defined a gene expression signature of PDGFR/ERK activation, we next sought to screen a library of small molecules to find compounds that would reverse the signature (for primary screen data, see Additional data file 1). We chose TIP5 fibroblast cells for the high-throughput screen instead of SH-SY5Y neuroblastoma cells used to define the gene expression signature. Both TIP5 and SH-SY5Y cells have wild-type PDGFR/ERK signaling, which makes it unnecessary to employ mutant and/or constitutively activated PDGFR cascades. TIP5 cells, however, were more adherent to 384-well plates, making them more amenable to the screening setting.

The screen was performed as follows. TIP5 cells were plated in 384-well plates, serum-starved overnight and compounds then added by pin transfer. The compound library, previously described in [2], consisted of 1,739 chemicals with previously established biological functions. Some of the compounds have been approved for use in humans by the Food and Drug Administration. After a 30 minute compound-incubation period, PDGF-BB was added. 45 minutes later, the growth medium was discarded, and cells were lysed. RNA was then extracted, the signature genes amplified by RT-PCR, and the PCR amplicons quantified by single-base extension mass spectrometry, as we previously described [2] (Figure 1c). Cells were treated in triplicate at two concentrations (approximately 10 μM and 50 μM). Compounds were defined as hits if the expression of two marker genes, *c-fos *and *EGR1*, normalized by expression of control genes was significantly (more than one standard deviation) lower than average expression in all positive control wells. Compounds that inhibited the signature of the activated PDGFR/ERK pathway in four out of six replicas were selected as hits for further characterization.

### Validation of hit compounds

**Figure 2:**
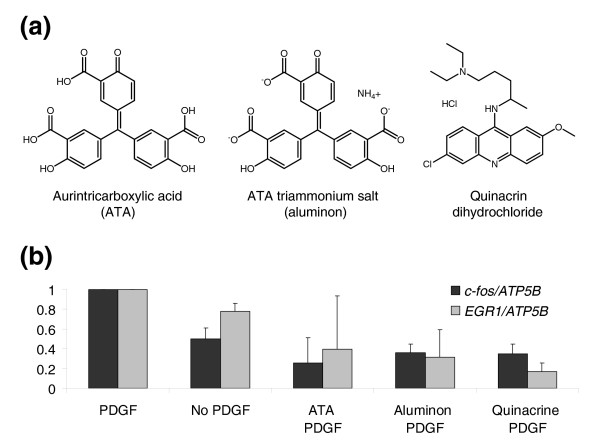
Hit compounds that passed hit selection criteria in the high-throughput screen. **(a) **Hit compounds identified in the screen. **(b) **High-throughput screen expression levels of marker genes *c-fos *and *EGR1*, normalized by control gene *ATP5B*, in the presence of 50 μM hit compounds and PDGF.

**Figure 3:**
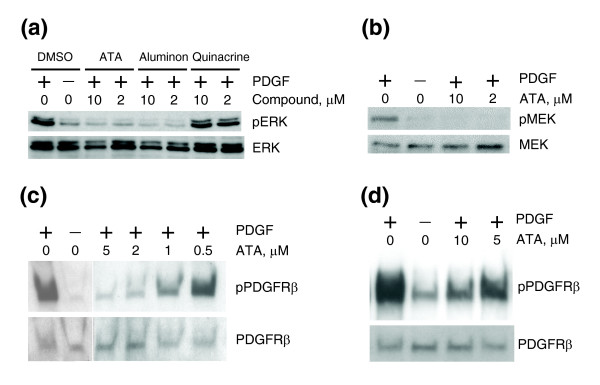
ATA abrogates phosphorylation of activated ERK, MEK and PDGFR. **(a) **ATA and aluminon, but not quinacrine dihydrochloride, abrogated PDGF-mediated phosphorylation of ERK. Western analysis of total TIP5 cell lysates. Cells were serum starved overnight and treated with ATA, aluminon, and quinacrine dihydrochloride in the presence of PDGF. pERK and ERK indicate antibodies against phospho-ERK and total ERK, respectively. DMSO, dimethyl sulfoxide. **(b) **ATA abrogates phosphorylation of MEK. Western analysis of total TIP5 cell lysates. Cells were serum starved overnight and treated with ATA and PDGF. pMEK and MEK indicate antibodies against phospho-MEK and total MEK, respectively. **(c) **ATA abrogates phosphorylation of PDGFR. Western analysis of total TIP5 cell lysates. Cells were serum starved overnight and treated with ATA and PDGF. pPDGFRβ and PDGFRβ indicate antibodies against phospho-PDGFRβ and total PDGFRβ, respectively. **(d) **Wash-out experiment: PDGFR phosphorylation remains inhibited upon removal of ATA. Western analysis of total TIP5 cell lysates. Cells were serum starved overnight and then incubated with ATA. After ATA was removed by washing, cells were induced with PDGF. pPDGFRβ and PDGFRβ indicate antibodies against phospho-PDGFRβ and total PDGFRβ, respectively.

Three wells met the hit selection criteria: aurintricarboxylic acid (ATA; free acid), aurintricarboxylic acid triammonium salt (aluminon), and quinacrine dihydrochloride (mepacrine) (Figure 2a,b); all three were therefore selected for further studies. Western analysis of total lysates from cells treated with these compounds demonstrated that both ATA and its salt (which in solution is identical to ATA), but not quinacrine dihydrochloride, abrogated PDGF-mediated phosphorylation of ERK (Figure 3a), thereby identifying ATA as an inhibitor of the ERK pathway. Quinacrine dihydrochloride did not inhibit ERK phosphorylation, but it has been previously shown to be a non-specific inhibitor of phospholipase A2 [14]. Activated ERK phosphorylates phospholipase A2 [15], and as a result stimulates transcription of the *c-fos *and *EGR1 *genes, two components of our ERK signature [16].

We then relaxed hit selection criteria, and identified nine more potential candidates. However, further study indicated that none of these nine additional compounds affected activation of the PDGFR/ERK pathway.

Disruption of phosphorylation of ERK by ATA was an indication that ATA inhibited the PDGFR/ERK pathway upstream of ERK. Subsequent analysis indicated that phosphorylation of both MEK (Figure 3b) and PDGFR (Figure 3c) was abrogated by ATA, thus pointing to PDGFR as a possible ATA target.

To address the possibility that ATA might in some fashion deplete PDGF ligand from the growth medium, TIP5 cells were first incubated with ATA for 30 minutes. Next, the cells were washed thrice with serum-free medium and then stimulated with PDGF. As shown in Figure 3d, PDGFR phosphorylation remained inhibited, suggesting that PDGF ligand was unlikely to be the target of ATA.

**Figure 4:**
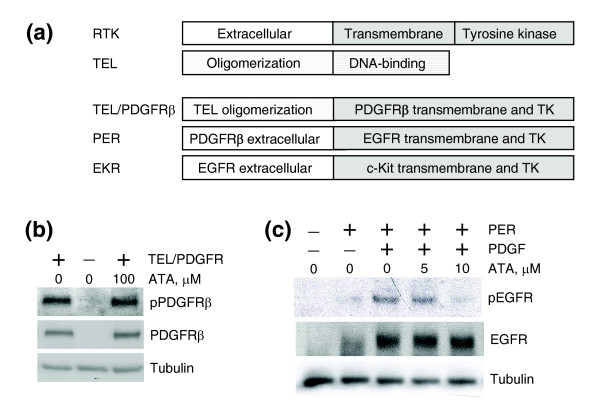
ATA targets the extracellular domain of PDGFR, not the transmembrane or cytoplasmic portions of the receptor. **(a) **Schematic representation of TEL/PDGFRβ, PER, and EKR. RTK, receptor tyrosine kinase; TK, tyrosine kinase. **(b) **ATA does not target the transmembrane or cytoplasmic portions of PDGFR. Western analysis of total lysates of Ba/F3 cells expressing TEL/PDGFRβ fusion protein. Cells were treated with ATA. pPDGFRβ, PDGFRβ, and tubulin indicate antibodies against phospho-PDGFRβ, total PDGFRβ, and total tubulin, respectively. **(c) **ATA targets the extracellular domain of PDGFR. Western analysis of total PER-PC12 cell lysates. Cells were serum-starved overnight and treated with ATA and PDGF. pEGFR, EGFR, and tubulin indicate antibodies against phospho-EGFR, total EGFR, and total tubulin, respectively.

The experiments described so far indicated that ATA inhibits PDGF-mediated ERK phosphorylation by inhibiting PDGFR phosphorylation. To localize the portion of PDGFR targeted by ATA, we utilized a series of chimeric receptor constructs (Figure 4a). The first chimera, TEL/PDGFR, is a naturally occurring, leukemia-associated fusion of the oligomerization domain of the transcription factor TEL (ETV6) to the transmembrane and cytoplasmic domains of PDGFR, resulting in constitutive activation of PDGFR [17]. As shown in Figure 4b, ATA was unable to inhibit TEL/PDGFR phosphorylation at concentrations as high as 100 μM, indicating that ATA does not target the transmembrane or cytoplasmic portions of PDGFR present in the TEL/PDGFR chimera.

**Figure 5:**
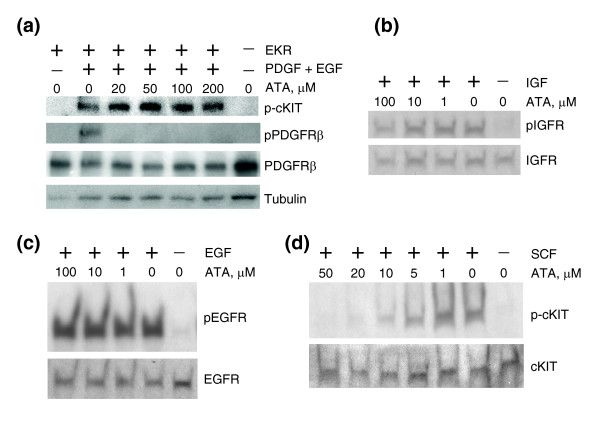
ATA failed to inhibit activated EKR, IGF1R, or EGFR, but inhibited SCF-mediated activation of cKIT. **(a) **ATA does not inhibit activated EKR. Western analysis of total TIP5 cell lysates. Cells were transfected with EKR plasmid, serum-starved overnight and treated with ATA, EGF and PDGF. p-cKIT, pPDGFRβ, PDGFRβ, and tubulin indicate antibodies against phospho-cKIT, phospho-PDGFRβ, total PDGFRβ, and total tubulin, respectively. **(b) **ATA does not inhibit activated IGF1R. Western analysis of total TIP5 cell lysates. Cells were serum starved overnight and treated with ATA and IGF. pIGFR and IGFR indicate antibodies against phospho-IGFR and total IGFR, respectively. **(c) **ATA does not inhibit activated EGFR. Western analysis of total TIP5 cell lysates. Cells were serum starved overnight and treated with ATA and EGF. pEGFR and EGFR indicate antibodies against phospho-EGFR and EGFR, respectively. **(d) **ATA inhibits SCF-activated cKIT. Western analysis of total MEL501 cell lysates. Cells were serum starved overnight and treated with ATA and SCF. p-cKIT and cKIT indicate antibodies against phospho-cKIT and total cKIT, respectively.

The next chimera, termed PER, is composed of the extracellular domain of PDGFR and the transmembrane and cytoplasmic domains of epidermal growth factor receptor (EGFR) [18]. ATA inhibited PER phosphorylation in PER-PC12 cells (Figure 4c), thus mapping the site of ATA action to the extracellular domain of PDGFR. To exclude the possibility of ATA inhibiting any receptor tyrosine kinase extracellular domain, we tested ATA against a third chimera, EKR, consisting of the extracellular domain of EGFR and the transmembrane and cytoplasmic domains of c-KIT [19]. ATA failed to inhibit EKR (Figure 5a), indicating that ATA exhibits some specificity for the PDGFR extracellular domain. Similarly, ATA failed to inhibit insulin-like growth factor (IGF)-induced phosphorylation of IGF1 receptor (IGF1R; Figure 5b), or EGF-induced phosphorylation of EGFR (Figure 5c) [20]. Interestingly, ATA did inhibit stem cell factor (SCF)-mediated activation of cKIT (Figure 5d). The cKIT and PDGFR extracellular domains have 41% sequence similarity (26% identity), whereas no significant homology is seen between the extracellular domains of PDGFR and EGFR or IGF1R.

**Figure 6:**
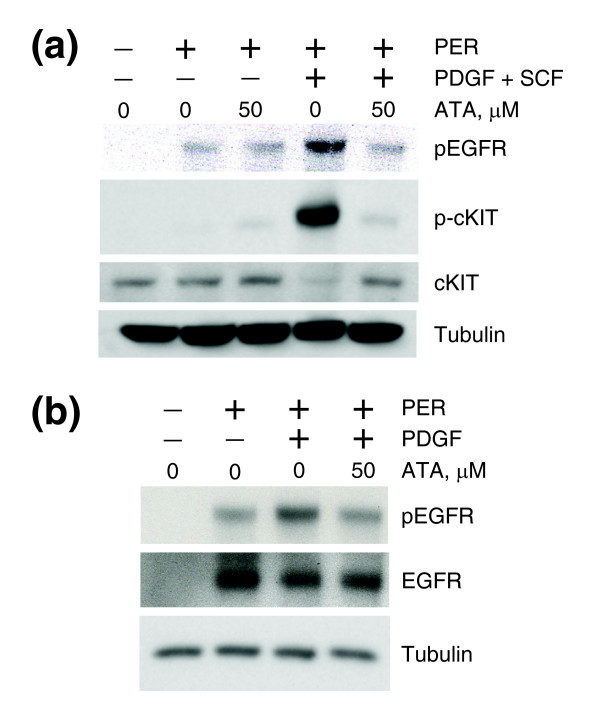
ATA does not fully abrogate phosphorylation of constitutively active PER. **(a) **Western analysis of total MEL501 cell lysates. Cells were transfected with PER plasmid, serum-starved overnight and treated with ATA, PDGF, and SCF. pEGFR, p-cKIT, cKIT, and tubulin indicate antibodies against phospho-EGFR, phospho-cKIT, total cKIT, and total tubulin, respectively. **(b) **Western analysis of total MCF7 cell lysates. Cells were transfected with PER plasmid, serum-starved overnight and treated with ATA and PDGF. pEGFR, EGFR, and tubulin indicate antibodies against phospho-EGFR, total EGFR, and total tubulin, respectively.

We note that whereas phosphorylation of the PER chimera is PDGF-dependent (and ATA inhibitable) in PER-PC12 cells, PER is constitutively active in 501 MEL and MCF7 cells, and in those contexts PER phosphorylation is not fully abrogated by ATA (Figure 6a,b). These experiments further point to the possibility of ATA inhibiting PDGF binding to the extracellular domain of PDGFR and disrupting ligand-mediated activation of the receptor.

### Structure-activity relationships in the series of ATA analogues

**Figure 7:**
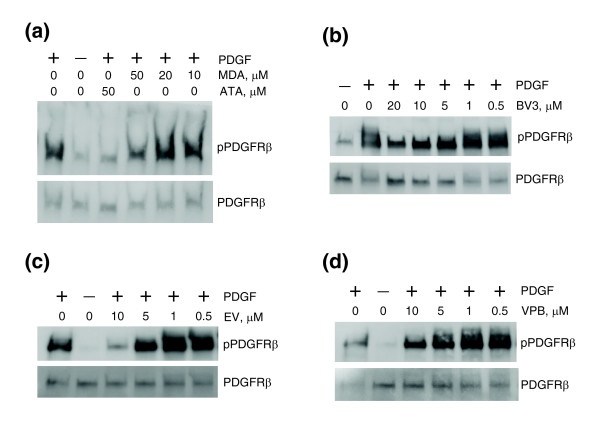
ATA analogues inhibiting PDGFR. Western analysis of total TIP5 cell lysates. Cells were serum starved overnight and treated with **(a) **5,5'-methylenedisalicylic acid (MDA), **(b) **Basic Violet 3 (BV3), **(c) **Ethyl Violet (EV), or **(d) **Victoria Pure Blue BO (VPB) and PDGF. pPDGFRβ and PDGFRβ indicate antibodies against phospho-PDGFRβ and total PDGFRβ, respectively.

In order to characterize the features of the ATA molecule required for biological activity, we analyzed a diverse set of ATA structural analogs (Figure S1 in Additional data file 2) available from the Available Chemicals Directory [21]. We split compounds into three groups to test three different hypotheses on the structure-activity relationship in the series. The activities of methylenedisalicylic acid, salicylic acid and 3-methylsalicylic acid (Figure S1a in Additional data file 2) were analyzed to examine if the skeletal-triphenylmethane structure of ATA was essential to its activity. Aurin, uranine and phenolphthalein sodium salt (Figure S1b in Additional data file 2) were tested to evaluate the roles the carboxyl and hydroxyl groups on the triphenylmethane scaffold play in the inhibitory potency of ATA. Compounds in the third group (Figure S1c in Additional data file 2) were evaluated to test the effect of various modifications of the phenyl rings on the inhibitory properties of ATA. No compounds in the series inhibited PDGFR at concentrations sufficient for ATA inhibition (less than 5 μM). In the first group, methylenedisalicylic acid (Figure 7a), but not methylsalicylic or salicylic acids inhibited PDGFR phosphorylation at 50 μM, suggesting that increasing the number of substituted salicylic acid moieties from one to three boosts the inhibitory potency of ATA. The positions and number of carboxyl and hydroxyl groups were essential for PDGFR inhibition, as indicated by the fact that no compounds in the second group inhibited PDGFR at 100 μM concentration. These results corroborate earlier reports that both the aurin triphenyl methane ring system and the carboxylic acid groups are necessary for ATA inhibitory properties [22].

**Figure 8:**
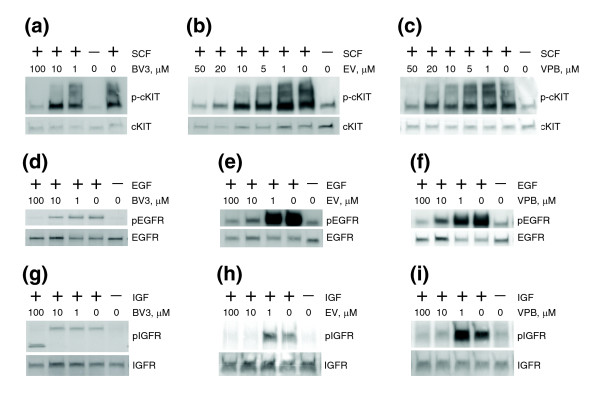
Basic Violet 3, Ethyl Violet, and Victoria Pure Blue BO exhibit less specific patterns of receptor inhibition than ATA. **(a-c) **Basic Violet 3 (BV3), Ethyl Violet (EV), and Victoria Pure Blue BO (VPB) inhibit SCF-activated cKIT. Western analysis of total MEL501 cell lysates. Cells were serum starved overnight and treated with BV3 (a), EV (b), or VPB (c) and SCF. p-cKIT and cKIT indicate antibodies against phospho-cKIT and cKIT, respectively. **(d-f) **BV3, EV, and VPB inhibit activated EGFR. Western analysis of total TIP5 cell lysates. Cells were serum starved overnight and treated with BV3 (d), EV (e), or VPB (f) and EGF. pEGFR and EGFR indicate antibodies against phospho-EGFR and total EGFR, respectively. **(g-i) **BV3, EV, and VPB inhibit activated IGF1R. Western analysis of total TIP5 cell lysates. Cells were serum starved overnight and treated with BV3 (g), EV (h), or VPB (i) and IGF. pIGFR and IGFR indicate antibodies against phospho-IGFR and total IGFR, respectively.

**Figure 9:**
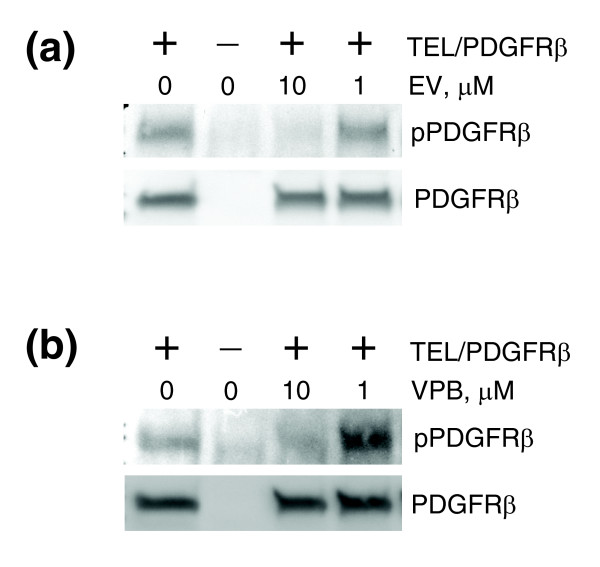
Ethyl Violet and Victoria Pure Blue BO inhibit cytoplasmic TEL/PDGFR. Western analysis of total lysates of Ba/F3 cells expressing TEL/PDGFRβ fusion protein. Cells were treated with either **(a) **Ethyl Violet (EV) or **(b) **Victoria Pure Blue BO (VPB). pPDGFRβ and PDGFRβ indicate antibodies against phospho-PDGFRβ and total PDGFRβ, respectively.

In the third group, Basic Violet 3, Ethyl Violet and Victoria Pure Blue BO inhibited PDGFR in the 5-10 μM range (Figure 7b-d). Interestingly, these three compounds exhibited less specific patterns of receptor inhibition than ATA, inhibiting not only cKIT, but also EGFR and IGF1R at 10-100 μM (Figure 8). Moreover, different from ATA, Ethyl Violet and Victoria Pure Blue BO readily translocated across the cell membrane, as indicated by their inhibition of cytoplasmic TEL/PDGFR in Ba/F3 cells at 10 μM (Figure 9). Taken together, these results suggest that the inhibitory mechanism of Basic Violet 3, Ethyl Violet and Victoria Pure Blue BO is different from the extracellular receptor inhibition mechanism of ATA.

## Discussion

In this report, we describe the proof-of-concept efforts to approach the discovery of inhibitors of signal transduction using a novel chemical genomic approach. We discovered a previously unknown property of the triphenylmethane derivative ATA, using GE-HTS. Having defined a signature of PDGFR activation, we screened a library of bioactive small molecules for compounds capable of turning off the signature. Importantly, the screen required neither a highly specialized signal transduction assay, nor prior knowledge of the protein to be targeted. In principle, small molecules acting upstream, downstream or at the level of PDGFR itself would be captured by the screen.

Two compounds in the library met pre-established criteria for hits abrogating the PDGFR/ERK activation signature. The hit compounds reproducibly inhibited the signature in follow-up studies, indicating that the false positive rate of the screen was quite low. One of the hits, quinacrine dihydrochloride, is a known inhibitor of phospholipase A2, a known regulator of ERK signaling [14-16]. The other compound, ATA, was a novel discovery, and was therefore followed up in greater detail.

ATA is a polymeric carboxylated triphenylmethane derivate with a molecular weight range of 422-6,500 [23], that has displayed a wide range of biological activity in *in vitro *biochemical assays. For example, ATA has been reported to inhibit enzymes involved in protein-nucleic acid interactions, including DNA and RNA polymerases, reverse transcriptase, nucleases, primases, topoisomerases, ribonucleotide reductases, aminoacyl-tRNA synthetase, nuclear factor-kappaB and HIV-1 integration protein [23]. In addition, ATA has also been shown to inhibit other classes of proteins *in vitro*, including phosphatases [24], NAD(H)/NADP(H)-requiring enzymes [25], aminopropyltransferases [26], mu- and m-calpain [27], delta aminolevulinic acid dehydratase [28], glucose-6-phosphate dehydrogenase [29], phenylalanine:tRNA ligase [30] and kinases, such as phosphofructokinase [31], ERK, p38 MAP kinase, IkappaB kinase [32], inositol-1,4,5-trisphosphate 3-kinase and inositol polypohosphate multikinase [33]. *In vitro *inhibition of protein synthesis has also been described [34].

Biological activity of ATA has also been observed *in vivo*, although in most cases only at rather high concentrations. For example, ATA is reported to obviate binding of interferon-alpha to its receptor in the 12-50 μM range [35], to prevent von Willebrand factor binding to platelet receptor glycoprotein Ib [36], and to block binding of gp120, the HIV coat protein, to its receptor, CD4 [23]. Similarly, ATA has been shown to be a N-methyl-D-aspartate (NMDA) receptor antagonist with an IC50 of 26.9 μM and was reported to antagonize excitotoxicity at both NMDA and non-NMDA glutamate receptors in the 50-100 μM range [37]. ATA inhibited progesterone receptor at 100-500 μM [38], estradiol receptor at 100-200 μM [39], and glucocorticoid receptor complex at 50-200 μM [23]. ATA also was reported to activate IGF1R (25-100 μM) [22] and erbB4 (10 μM) [40]. These studies suggest that ATA has a range of biological activities, most of which, however, are observable only at quite high concentrations, in many cases as high as 100 μM.

More limited activity has been reported at lower concentrations of ATA. For example, at 1-5 μM, ATA was reported to reverse the transformed phenotype of cells transfected with basic fibroblast growth factor fused to a signal peptide sequence (spbFGF cells) [41]. It was suggested, on the basis of ATA fluorescence studies, that ATA binds to acidic fibroblast growth factor, altering its physicochemical properties and decreasing its mitogenic activity [42], although these results were not confirmed by more direct biochemical methods. The observed ATA interactions in this setting take place at the cell surface, consistent with the finding that ATA does not readily penetrate cellular membranes. ATA is not taken up by HeLa cells, VERO cells, rabbit reticulocytes, or a variety of bacterial cells [43]. Accordingly, ATA did not inhibit intracellular proteins, even at concentrations hundreds of times higher than those required for inhibition *in vitro *[37]. Only at high concentrations (500 μM) was intracellular ATA fluorescence detectable [24]. It seems most likely, therefore, that our observed effects of ATA on PDGFR activity occur at the cell surface.

Consistent with this notion, our analysis indicated that all signaling downstream of PDGFR was inhibited by ATA, and ATA wash-out experiments suggested that ATA did not abrogate the signaling by binding and inactivating PDGF. Furthermore, analysis of chimeric PDGFR constructs localized the ATA effect to the PDGFR extracellular domain. Interestingly, modest concentrations of ATA (2-5 μM) also inhibited activity of the related receptor tyrosine kinase cKIT, which shares sequence homology with PDGFR in the extracellular domain, whereas kinases lacking such homology (for example, IGFR and EGFR) were inhibited only at concentrations of 100 μM. It is possible that the previously described inhibition of JAK/STAT signaling by ATA [32,44] is attributable to its inhibition of PDGFR family receptor tyrosine kinases, known to be upstream activators of the JAK/STAT pathway [45,46].

## Conclusion

The polymeric nature of ATA may make it unattractive as a therapeutic agent and, moreover, multiple highly potent PDGFR kinase inhibitors have been previously reported [47]. Our work establishes proof of concept, however, for the notion that mRNA expression signatures can be effectively used as a read-out for the identification of inhibitors of signal transduction, often thought approachable only through the direct examination of protein phosphorylation states. We note that indeed antibody-based high-throughput screens have been reported [48], but such assays obviously require the availability of a sufficiently sensitive and specific antibody for this purpose. For many, if not most, proteins of interest, such high quality antibodies are not available. The ability to convert any biological process or cell state into a completely generic gene expression signature that can be monitored in high throughput and at low cost is therefore attractive.

The implementation of the GE-HTS concept described here involves the detection of multiplexed RT-PCR signature genes by a single-base-extension reaction followed by MALDI-TOF (matrix assisted laser desorption ionization-time of flight) mass spectrometry [2]. While this method was effective in the study described here, it has several limitations. For example, conventional RT-PCR amplification is not easily multiplexed, and the ability to simultaneously detect multiple amplicons by the mass spectrometric method is limited. Lastly, the approach can become expensive if extended to the ultra-high-throughput setting. We have therefore modified the approach to allow for the efficient amplification of up to 100 transcripts using a ligation-mediated amplification method, followed by detection on polystyrene beads via flow cytometry, as we recently described [3,5,49]. The present study, however, establishes that the GE-HTS concept can be applied to screening for modulators of signal transduction, representing a general approach to the discovery of compounds that affect any signaling pathway of interest.

## Materials and methods

### Reagents

The EKR construct [19] was kindly provided by Dr. Ullrich, Department of Molecular Biology, Max-Planck-Institut fur Biochemie. PER chimera [18] was a gift of Dr. Tyson and Dr. Bradshaw, Department of Physiology and Biophysics, University of California, Irvine.

Chemical compounds apigenin, U0126, quinacrine dihydrochloride and ATA were obtained from Calbiochem [50]; Methyl Violet B base, Rhodamine 6 G tetrafluoroborate, sulforhodamine, Ethyl Violet, Victoria Pure Blue BO, Rhodamine B, Lissamine Green B, Methyl Violet 2B, Rhodamine 6G, (L-Asp)2Rhodamine 110 TFA, Rhodamine 110 chloride, Eosin B, Rhodamine 123 hydrate, Rhodamine 19 perchlorate, Acid Fuchsin calcium salt, p-Rosolic acid, Basic Violet2, Gentian Violet, pararosaniline hydrochloride, and salicylic acid were purchased from Sigma [51]; and 3-methylsalicylic acid, 5,5'-methylenedisalicylic acid, phenolphthalein sodium salt, and Uranine K were obtained from ABCR [52].

Growth factors PDGF, EGF, and SCF were obtained from Cell Signaling [53], R^3^IGF from Sigma, and interleukin (IL)3 from R@D Systems [54].

Cell culture reagents RPMI 1640, Dulbecco's modified Eagle's medium (DMEM), and HAM's F-10 were purchased from Mediatech [55], penicillin and streptomycin from Invitrogen [56], and fetal bovine serum from Sigma. p44/42 MAP kinase, phospho-p44/42 MAP kinase (Thr202/Tyr204), MEK1/2, phospho-MEK1/2 (Ser217/221), PDGF-BB, phospho-PDGFRβ (Tyr751), phospho-EGFR (Tyr1068), cKIT, Phospho-cKIT (Tyr719), IGF-IαR, and Phospho-IGF-IR (Tyr1131)/insulin receptor (Tyr1146) antibodies were obtained from Cell Signaling. EGFR and mouse cKIT antibodies were purchased from Santa Cruz Biotechnology [57]. Alfa-tubulin antibody was obtained from Sigma.

### Cells

SH-SY5Y neuroblastoma cells were purchased from American Type Culture Collection [58]. The IL3-dependent pro-B lymphoid cell line Ba/F3 and Ba/F3 cells expressing TEL/PDGFRβ [17,59] were obtained from Dr. Gary Gilliland. TIP5 primary fibroblasts [60] were a gift from Dr. Stephen Lessnick. We thank Dr. Ruth Halaban for 501 MEL human melanoma cells. PER-expressing PC12 cells were generously provided by Dr. Darren Tyson. SH-SY5Y, PC12, TIP5 and MCF7 cells were cultured in DMEM, BaF3 cells and BaF3 cells expressing TEL/PDGFRβ were maintained in RPMI 1640 medium, and 501 MEL cells were grown in Ham's 10 medium. Medium for IL3-dependent Baf3 cells was supplemented with 0.05 ng/ml IL3. Media for all cell lines except PC12 contained 10% fetal bovine serum, 10 U/ml penicillin, and 10 μg/ml streptomycin. PC12 cells were grown in DMEM with 15% horse serum, 5% fetal bovine serum, 10 U/ml penicillin, and 10 μg/ml streptomycin. All cells were grown at 37°C in 5% CO_2_.

### Characterization of the activation signature for ERK/PDGFR pathway

SH-SY5Y cells were grown to confluence and starved overnight in serum-free medium in order to silence any sustained effects from growth factor signaling. Prior to induction with 50 ng/ml PDGF, cells were treated with pathway inhibitors 74 μM apigenin or 50 μM U0126, or with dimethyl sulfoxide (DMSO) as carrier for 60 minutes. Total RNA was isolated 30 minutes after PDGF addition. Experiments were performed in duplicate. The RNA was processed and hybridized with Affymetrix U133A GeneChips as described in [61].

To define the ERK/PDGFR activation signature, a pair-ranking algorithm was used. Genes were ranked according to their raw expression values on each chip. Ten genes with maximum change in ranking were selected for each one of three pairs of conditions: cells with PDGF versus cells without PDGF, cells with PDGF versus cells with PDGF and apigenin, and cells with PDGF versus cells with PDGF and U0126. Three genes common to all three conditions were selected as a signature of the activated ERK/PDGFR pathway. The signature was then trimmed from three to two genes based on their relative strength of expression in TIP5 cells.

### Screening methods

TIP5 cells were grown to confluence and starved overnight with 20 μl serum-free medium per well of 384-well plates. We added 20 μl of compounds diluted in media so that the final concentration of compounds would be approximately 10 μM in three out of six replicas, and 50 μM in three remaining replicas. Media containing carrier (DMSO) was added to control wells instead of compounds. The compound library was composed of 1,739 chemicals either approved for use in humans by the Food and Drug Administration or extensively biologically characterized [2,62] (the full list of tested compounds is available in these publications). After 30 minutes of compound treatment, cells were induced with 40 μl of PDGF diluted in media (final PDGF concentration 40 ng/ml). PDGF was added to half of control wells to measure PDGF response; only media was added to the remaining control wells. After 40 minutes of PDGF induction, media was discarded, cells were lysed and RNA was extracted and quantified as described in [2].

Briefly, 15 μl of lysis solution containing a hypotonic detergent, dithiothreitol and RNAse inhibitor were added to medium-free cells for 15 minutes. The lysates were transferred to a 384-well oligo-dT-coated plate and incubated with 6 μl of 2.5× binding buffer. After 30 minutes of incubation lysates were discarded and reverse transcription was carried out in a 5 μl Moloney murine leukemia virus (M-MuLV) RT reaction at 37°C for 2 h.

After incubation, the RT mixture was discarded and multiplex PCR was carried in a 5 μl volume. The resulting mixture was treated with shrimp alkaline phosphatase and the single-base extension reactions were carried out in 9 μl reaction volumes with 1× Thermosequenase buffer, 2.7 μM of each primer, 0.2 mM of each ddNTP and 0.58 units per reaction of Thermosequenase as described in [2].

The lysis buffers, 384-well custom-coated oligo-dT plates, and M-MuLV were purchased from Pierce [63] and were used according to a modified version of the Express Direct mRNA Capture and RT-PCR system. The shrimp alkaline phosphatase, Thermosequenase buffer, ddNTP and Thermosequenase were obtained from Sequenom [64].

The primers used for multiplex PCR reactions were: *EGR1*, 5'-AGC GGA TAA CAC CTC ATA CCC ATC CCC TGT-3' and 5'-AGC GGA TAA CTG TCC TGG GAG AAA AGG TTG-3'; *c-fos*, 5'-AGC GGA TAA CGC TTC CCT TGA TCT GAC TGG-3' and 5'-AGC GGA TAA CAT GAT GCT GGG AAC AGG AAG-3'; *ATP5B*, 5'-AGC GGA TAA CCA AAG CCC ATG GTG GTT ACT-3' and 5'-AGC GGA TAA CGC CCA ATA ATG CAG ACA CCT-3'; *RPL23A*, 5'-AGC GGA TAA CAA GAA GAA GAT CCG CAC GTC-3' and 5'-AGC GGA TAA CCG AAT CAG GGT GTT GAC CTT-3'.

The following primers were used for single-base extension reactions: *EGR1*, 5'-TTC CCC CTG CTT TCC CG-3'; *c-fos*, 5'-TGC CTC TCC TCA ATG ACC CT-3'; *ATP5B*, 5'-GAC TGT GGC TGA ATA CTT CA-3'; *RPL23A*, 5'-GTC TGC CAT GAA GAA GAT AGA A-3'.

To select compounds that inhibited expression of the pathway signature, the following procedure was performed. For each compound on each plate four ratios were determined: expression of *EGR1 *versus expression of *ATP5B*(V_EGR1/ATP5B_), *EGR1 *versus *RPL23A *(V_EGR1/RPL23A_), *c-fos *versus *ATP5B *(V_c-fos/__ATP5B_), and *c-fos *versus *RPL23A *(V_c-fos/RPL23A_). For each plate a median (μ) and standard deviation (σ) were determined for each of four ratios. A compound was considered a plate hit if each of the four ratios for this compound were at least one standard deviation smaller than the median (V < (μ - σ): V_EGR1/ATP5B _< μ_EGR1/ATP5B _- σ_EGR1/ATP5B_; V_EGR1/RPL23A _< μ_EGR>1/RPL23A _- σ_EGR1/RPL23A_; V_c-fos/ATP5B _< μ_c-fos/ATP5B _- σ_c-fos/ATP5B_; and V_c-fos/RPL23A _< μ_c-fos/RPL23A _- σ_c-fos/RPL23A_). Compounds that were plate hits in four out of six replicas were selected for further consideration.

### Western blotting and transfection

For transfection experiments, 501 MEL cells or TIP5 cells were grown overnight to 50% confluence and transfected using Fugene 6 transfection reagent (Roche [65]) as recommended by the manufacturer. Then 24 h after transfection, medium was exchanged for a serum free one, and cells were serum starved overnight.

Otherwise, adherent cells (TIP5, MEL 501) were grown to confluence, serum starved overnight, and treated with compounds and growth factors as indicated. Cells growing in suspension (BaF3 cells and BaF3 cells expressing TEL/PDGFR protein) were grown to 10^6 ^cells/ml and treated with compounds as indicated. After treatment media was removed, adherent cells were scraped with Sample Buffer from Cell Signaling, and suspension cells were pelleted and resuspended in Sample Buffer. The resulting lysates of approximately 1 × 10^5 ^cells were boiled, chilled, run on 4-15% gradient gels from BioRad [66], transferred to a polyvinylidene difluoride membrane from Millipore [67], blocked, probed and visualized as recommended by the antibody manufacturers.

### Sequence alignment

Comparative sequence analysis between PDGF (UniProtKB/Swiss-Prot entry P09619), cKIT (UniProtKB/Swiss-Prot entry P10721), EGFR (UniProtKB/Swiss-Prot entry P00533), and IGFR (UniProtKB/Swiss-Prot entry P08069) was performed with BLAST 2 SEQUENCES [68].

### Averaging and normalization of high-throughput screen expression levels of marker genes *c-fos *and *EGR1 *for Figure 2b

Each primary screen replica plate contained 16 wells with PDGFβ and carrier DMSO as a positive control for PDGF activation (called PDGF in Figure 2b), and 16 wells with carrier only as a negative control for PDGF activation (called No PDGF in Figure 2b). The expression levels of marker genes normalized by expression of control gene (ratios *c-fos*/*ATP5B *and *EGR1*/*ATP5B*) were averaged for 16 PDGF wells to have a single value for the positive PDGF control per plate, and for 16 No PDGF wells to have a single value for the negative No PDGF control per replica plate. Only one well was allocated for each hit compound on a single replica plate.

To compare data between replica plates in Figure 2b, the ratios *c-fos*/*ATP5B *and *EGR1*/*ATP5B *were adjusted to be equal to 1 for positive PDGF control. This means that on each replica plate the marker/control ratios in all wells were divided by the corresponding value for the positive PDGF control for this plate. The procedure was performed independently for both *c-fos*/*ATP5B *and *EGR1*/*ATP5B *ratios. As a result of this procedure, the *c-fos*/*ATP5B *and *EGR1*/*ATP5B *ratios for the hit compounds and for the No PDGF control on each plate were divided by PDGF control *c-fos*/*ATP5B *and *EGR1*/*ATP5B *ratios for this plate. The resulting adjusted values were then averaged between three replica plates.

### Data

The data have been deposited in the Gene Expression Omnibus [69] with accession number GSE7403.

## Abbreviations

ATA, aurintricarboxylic acid; DMEM, Dulbecco's modified Eagle's medium; DMSO, dimethyl sulfoxide; EGF, epidermal growth factor; EGFR, EGF receptor; ERK, extracellular regulated kinase; GE-HTS, gene expression-based high-throughput screening; IGF, insulin-like growth factor; IGF1R, IGF1 receptor; IL, interleukin; MAP, mitogen activated protein; MEK, mitogen/extracellular signal-regulated kinase; M-MuLV, Moloney murine leukemia virus; NMDA, N-methyl-D-aspartate; PDGF, platelet derived growth factor; PDGFR, PDGF receptor; RT, reverse transcriptase; RTK, receptor tyrosine kinase; SCF, stem cell factor.

## Authors' contributions

AA designed, performed and analyzed experiments, and drafted the manuscript. BS guided experimental design, provided chemical libraries, and assisted in writing of the manuscript. TG supervised the design and execution of experiments, and participated in writing of the manuscript.

## Additional data files

The following additional data are available. Additional data file 1 contains primary screen data archived in WinZip format. Additional data file 2 contains Figure S1 showing ATA analogs.

## Supplementary Material

Additional data file 1 Primary screen data. Click here for file

Additional data file 2 ATA analogs. Click here for file
