# Supplementary material for: Gene expression-based screening for inhibitors of PDGFR signaling
Source: Genome Biol. 2008 Mar 1;9(3):R47. doi: 10.1186/gb-2008-9-3-r47 (PMC2397499; doi:10.1186/gb-2008-9-3-r47)
Supplement: Additional data file 2 — ATA analogs. [file gb-2008-9-3-r47-S2.pdf]

A

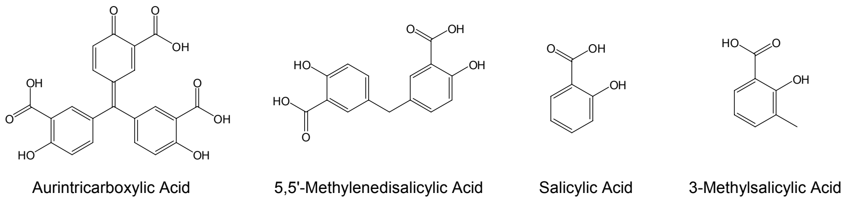
Aurintricarboxylic Acid
5,5'-Methylenedisalicylic Acid
Salicylic Acid
3-Methylsalicylic Acid

B

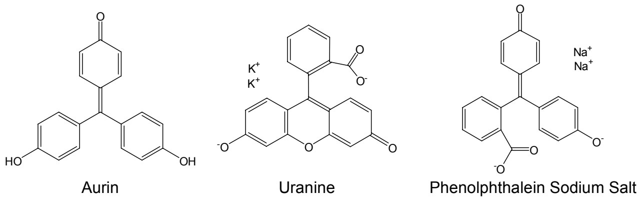
Aurin
Uranine
Phenolphthalein Sodium Salt

C

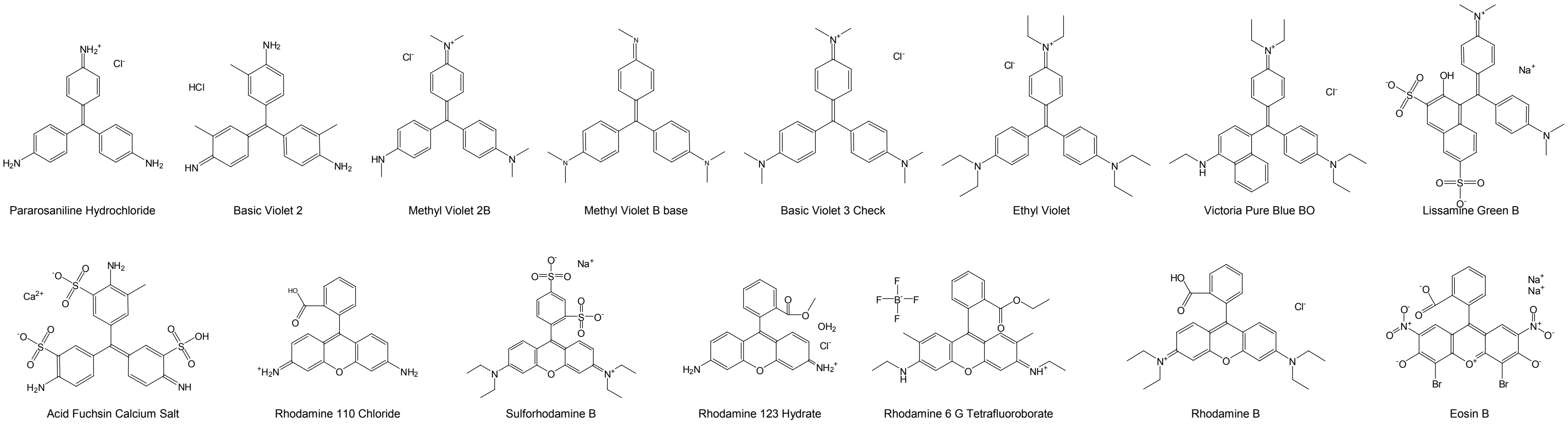
Pararosaniline Hydrochloride
Basic Violet 2
Methyl Violet 2B
Methyl Violet B base
Basic Violet 3 Check
Ethyl Violet
Victoria Pure Blue BO
Lissamine Green B
Acid Fuchsin Calcium Salt
Rhodamine 110 Chloride
Sulforhodamine B
Rhodamine 123 Hydrate
Rhodamine 6 G Tetrafluoroborate
Rhodamine B
Eosin B

Figure S1. ATA analogs. A. Compounds used to test importance of the skeletal-triphenylmethane structure of ATA for its activity. B. Compounds used to evaluate the role the carboxyl or hydroxyl groups. C. Compounds used to test the effect of various modifications of the phenyl rings on the inhibitory properties of ATA.
